# Effects of blinded and nonblinded sequential human redundancy on inspection effort and inspection outcome in low prevalence visual search

**DOI:** 10.1038/s41598-024-72210-8

**Published:** 2024-10-03

**Authors:** Dietlind Helene Cymek

**Affiliations:** Department of Psychology and Ergonomics, Technische Universität Berlin, Berlin, Germany

**Keywords:** Human behaviour, Risk factors

## Abstract

Human redundancy is often used in safety–critical domains to help protect against errors. For example, mammograms are read by two radiologists, or the dose of a drug is calculated by two physicians who perform the task redundantly one after the other. However, the expected reliability gain may be compromised by social loafing (SL), i.e., a reduction of individual effort caused by the team setting. In two laboratory studies, we therefore investigated whether different forms of human redundancy cause SL and impact task outcome. In each experiment, about 20 participants inspected images alone for rare targets, while 40 participants worked in teams of two, either performing the first or second inspection. We measured effort using inspection time and inspection area, and measured task outcome using the number of detected targets and false alarms. While there was no transfer of target marks in experiment 1 (blinded redundancy), the second inspectors in experiment 2 saw marks set during the first inspection (nonblinded redundancy). In experiment 1, data revealed no indications of SL but increased time, area covered, detections and false alarms for team-based relative to solo performance. In experiment 2, teams clearly adapted their inspection intensity. The second inspectors searched the images significantly shorter than the first inspectors. While detected targets did not differ between the two redundant groups, the second inspectors were found to produce significantly fewer false alarms than the first inspectors. However, the advantage of detected targets and area coverage still favored the teams. Thus, the principle of redundancy was effective in both forms of sequential redundancy, even if it led to different behaviors and perceptions at the individual level.

## Introduction

This article focuses on visual search tasks in low prevalence contexts such as mammography and baggage screening. Here, the search task is difficult, and the targets are extremely rare (e.g., 3–5 cancers per 1000 breast cancer screening cases)^1–3^. Unfortunately, previous research has found that if you don't find it often, you often don't find it^4–6^ and has termed this phenomenon the low-prevalence effect. Because misses, such as undetected cancers or knives, are potentially costly in these search tasks, a lot of effort is put into making detection as reliable as possible. For example, screeners are trained extensively and are supported by increasingly sophisticated software (computer-assisted detection; CAD)^7–12^. Another approach is to increase the prevalence of targets artificially. In baggage screening, for example, the checkpoint X-ray places fake threat images into the images, a measure known as threat image projection (TIP), to increase the detection of extremely rare real threats^4^. Furthermore, the task is performed by not just one but by two screeners, because four eyes presumably see more than two^13–16^.

This latter measure is called human redundancy and is frequently used in safety–critical industries, to ensure high levels of task or process reliability. The advantage of redundancy is not only the combination of different people with possibly different skills but also the fact that more time is invested in the task overall, which on many tasks leads to better performance. The redundancy principle is for example common on aircraft and large ships with advanced automation, where two or more people are present to monitor the autopilot and take action if the system fails. Because everyone is working on the task at the same time, this form of redundancy is called parallel redundancy^17,18^. Another type of redundancy is sequential redundancy, where people work on the task one at a time in a sequential order^19–21^. For example, at an airport, one screener examines the X-ray scans of carry-on bags and flags any suspicious item that needs further examination by another screener. Similarly, in hospitals, one nurse determines a patient's medication dose first and then a second nurse does it again to avoid incorrect dosing^22^, and in mammography screening, two radiologists read the same X-rays to improve breast cancer detection^15^. Sometimes the diagnoses or calculated drug doses of the first person performing the task are made available to the second person, sometimes they are not. No matter which variant of redundancy is used, they are all in place to ensure that operations are fail-safe. If one person is not completely reliable or is biased in some way (e.g., a radiologist missing a cyst in a mammography screening), the other person can compensate for this suboptimality by complementing or correcting their behavior.

Theoretically, if both people working redundantly were completely independent, the gain in reliability (e.g., detection of rare targets) could be greatly increased. For example, if one person's reliability to identify a suspect component is 70.0% ([1(0,3)] × 100), a second person with similar skills working completely independently could increase the team's joint reliability to identify that component to 91.0% ([1−(0.3)^2^] × 100), and theoretically a third person would further increase the reliability to 97.3% ([1 − (0.3)^3^] × 100). In practice, however, people who work redundantly can rarely be considered completely independent, making it difficult to judge how much reliability can be gained by adding a second person. In most work settings where human redundancy is used, the people involved are usually aware that the task is being done twice and that they are fulfilling a backup role. According to socio-psychological research in the field of group work, this awareness could alter their behavior and decision-making in many—sometimes unconscious—ways. For example, the mere presence of another person has been found to increase individual effort and task outcome in simple and well-trained tasks—a phenomenon called social facilitation^23,24^. More effort and better results can also occur in a group setting when group members try to outperform each other on tasks for which individual contributions are recognizable^25^. Furthermore, social compensation can occur in cases where a person tries extra hard to compensate for a team member who is not performing as well^18,26^.

However, in settings where two or more individuals perform the same task redundantly, the group setting can also reduce individual effort and task outcome, as in the case of social loafing^27–29^. Social loafing (SL) is defined as lower individual effort put into a task performed in a team than on a task performed alone^30^. For example, individuals have been found to clap more quietly or pull less hard on a rope when doing it with others than when doing it alone^28,31^. SL is strongly associated with reduced identifiability of individual contributions and reduced evaluation potential in teamwork, leading to a decrease in motivation^30^. This effect is further moderated by factors such as task valence, peer performance expectations, and uniqueness of individual task contributions^30^. Specifically, SL is higher when evaluation potential is low^27,28,32–34^, when a coworker performs well on the task^26,27^, and when group members' task contributions are redundant^35^. Recent research suggests that social loafing is even a topic in human–robot teams^36,37^, indicating that social team effects may not be limited to human-only teams but might be relevant when humans are working with technological partners also.

Despite the theoretical possibility of SL effects occurring in human redundancy settings, very little experimental research is available. Furthermore, most of the research in this area has addressed the case of parallel redundancy, comparing two people who work simultaneously on a task (e.g., monitoring and cross-checking an automated system) with people working alone^17,18,38,39^. Two studies in the context of aviation compared the number of detected autopilot failures of two-person teams with single individuals and found no team advantage^38,39^. These studies speculated that individuals in the team setting might have reduced their effort to crosscheck the autopilot (i.e., SL) and as a consequence did not detect more failures than people working alone. Other studies of parallel human redundancy directly measured the effort invested in a task to identify SL^17,18^. These studies directly tracked the cross-checking behavior performed to verify automated decisions in a chemical plant and found that people did indeed reduce their cross-checking behavior when working in a team of two compared to when working alone^17,18^. One of these studies even found that SL was so substantial that the joint cross-checking of teams (number of automated decisions cross-checked by at least one of the two team members) did not significantly differ from that of individuals working alone, calling into question the effectiveness of the redundancy principle for the monitoring task studied^17^.

But what about redundant tasks that are performed sequentially rather than in parallel? Can SL occur in double checks in mammography and baggage screening, or when medication doses are determined redundantly? And what are the effects of providing inspection results of the first person to the second person in line? There are some indications of SL from qualitative research in the healthcare sector. A couple of interview studies, for example, claimed that double-checking medication dosage^22,40^ comes with the risk of reduced individual responsibility and effort, the neglect of this process under time pressure, and the tendency to defer to authority^22^. This is a very clear indication that SL can occur. Furthermore, there are multiple studies in health care that investigated the effectiveness of double reading in mammography^16,41–43^. However, while some studies found a small team advantage in cancer detection over solo inspection^15,44^, others could not find a difference^16^, or reported that the increase in detected cancers was outweighed by high false-alarm rates^41^. Another study investigated whether a higher detection rate could be achieved with or without the transmission of the diagnostic result of the first radiologist to the second radiologist (blinded vs. nonblinded redundancy) and found a marginal detection advantage of blinded redundancy but also to the expense of slightly more false alarms^13^. While the lack of an obvious team advantage in cancer detection could again be theoretically caused by a reduced individual effort invested in the medical assessment when redundancy is used, this remains speculative until direct measures of effort, such as search duration or eye tracking data, provide clarification. However, since all these studies were not designed to detect SL, they did not differentiate between the two positions in the sequence, nor did they use direct measures of effort.

There are only a few studies that investigated sequential human redundancy from a socio-psychological perspective. One was a laboratory study, which compared the time spent checking a document (effort measure) and the errors found in the document (outcome measure) in individual and teamwork^19^. The study found that when a highly reliable person had already completed the review, the second person spent less time and missed more errors (i.e., social loafing). However, this was not the case when a less reliable person was performing the task first.

Another set of studies investigated SL in sequentially redundant nondestructive testing (NDT) of materials^21^. In a simulated NDT task, participants searched for discontinuities (e.g., cracks, pores, inclusions) in images of a copper component obtained by ultrasonic testing. A first study investigated whether SL occurs when a first inspector assumes that a second inspection will follow but found no difference in the measured detection rate (outcome measure) of first inspectors compared to a solo condition. A second study compared two groups. One group consisted of second inspectors who were informed that they would be working with a highly experienced colleague, and the other group consisted of second inspectors who were only informed that they would be working with another person. According to social loafing theory, the expectation of a high performing colleague makes social loafing more likely^26,27^. In the experiment, however, only 66% of the discontinuities were detected by the first inspector. Thus, both groups learned that the first inspector was in fact only moderately reliable and that they could make a significant contribution to the team's performance. The study found no difference in target detection between the two groups (i.e., no indication of SL), suggesting that the actual experience of coworkers' errors was stronger than the general a priori information that a coworker is highly experienced.

The third and more recent set of studies that investigated SL in sequential human redundancy was performed in the context of chemical production^20^. In two laboratory experiments, participants had to perform three tasks in parallel, one of which was a quality-control task that was either performed alone or with sequential redundancy. The quality-control task was realized in different experiments either in a blinded or in a nonblinded manner, operationally defined by whether or not evaluations of the first team member were forwarded to the second one. SL was expected in both inspection positions in the blinded experiment and was expected to be particularly pronounced in the second position in the nonblinded experiment, where participants (those in the second position) received the quality control ratings of a highly reliable team partner working in position 1. In position 1 in the nonblinded experiment, however, no SL was expected due to the higher evaluation potential caused by the transfer of quality-control decisions to position 2. Results did not reveal SL in the blinded setting for either position^20^. In the nonblinded setting, team members working in the second position, as expected, substantially reduced their verification effort (without increasing the effort invested in the parallel task they were alone responsible for), while no SL was found in the first position. The different amount of effort invested by the three groups was also reflected in the detection rates of quality deficiencies. These studies suggest that sequential human redundancy can lead to SL when a nonblinded process is used but is less of a problem when a blinded process is used. In both experiments teams checked jointly more chemicals and detected more quality defects than participants working alone, showing that human redundancy despite some losses led to more safety.

But are these results obtained in a multitasking setting transferable to low-prevalence visual search context where no parallel tasks need to be performed? In other words, are these results also relevant to mammography and baggage screeners? Can SL occur there and to what extent?

Theoretically, it is possible that the SL effects found in the multitasking study earlier^20^ were specifically influenced by the multitasking. Multitasking requires balancing different task demands and constant switching between tasks and is therefore often associated with higher workloads. Although SL theory does not make any assumptions about the effect of competing tasks and workload, it could be speculated that people show more SL when their workload is high. At least when working with technological partners, it has been shown that individual effort in a team task is reduced more when the workload is high^45^. Another study found that errors made by a technical partner are detected less often in multitasking than in single-task settings^46^. Based on these findings, it might be expected that in single-task settings, where the workload tends to be lower than in multitasking, the tendency to engage in social loafing is reduced as well.

However, SL is more likely to occur in simple than in difficult tasks, since participants in difficult tasks feel that their contributions are not simply duplicated and that their contributions are needed^35^. In a multitasking setting (where the overall task difficulty is generally high), team members may assume that their team partner cannot always perform all tasks perfectly and that their contribution is needed. In a single-task setting, however, this may be less the case, which potentially could encourage SL.

Redundant checks can also differ regarding another aspect, that might increase the strength of an SL effect. In the multitasking study^20^, there was only a general alert that a chemical product was deficient, without specifying the concrete deviating parameters. To verify the alert of a team partner, all parameters needed to be checked. However, such a global alert is atypical for baggage and mammography screening. As soon as a radiologist or baggage screener detects an anomaly, it is marked locally. This allows position 2 to easily verify the result of position 1 (in the case of nonblinded sequential redundancy) by examining only the locally marked image area and giving less attention to unmarked areas. The use of local markers can therefore theoretically strengthen the SL effects in this scenario.

In summary, certain aspects of sequential redundancy in baggage and X-ray screening can theoretically either increase or decrease SL compared to the chemical control work tasks used in previous studies. This makes predictions more difficult. Nevertheless, we assumed that SL would also occur in the single task—firstly, because SL was already found during monotasking when checking documents for errors^19^, and secondly, because the SL effect found in the multitasking study (nonblinded, position 2) was very large^20^. Therefore, we assumed that the previously found SL effects^20^ in blinded and nonblinded sequential human redundancy were transferable to single-task settings and the application context of baggage and mammography screening.

To test our assumption, we conducted two experiments analogous to these latest studies of sequential human redundancy^20^, but with a single-task setup. We used a visual inspection task, simulating mammography readings to make the task more representative of redundancy tasks encountered in the field. For this purpose, local target marks were used, and slight time pressure was induced, as is usual in the area of mammography and baggage screening. The first experiment compared people working alone to people working sequentially redundant in a blinded manner, and the second experiment compared people working alone with individuals working sequentially redundant in a nonblinded manner. In sum, these studies aimed to investigate, whether SL occurs in a visual search task that is more representative of the sequentially redundant tasks commonly used in practice. Specifically, we aimed to answer whether it makes a motivational difference to be first or second in line to work on a visual inspection task and whether the decision or work outcome of the first team member should be transferred to the second team member in line or not.

Furthermore, we took measures of subjective effort to exploratively investigate whether SL is a conscious or unconscious process. Previous research on SL has not reached a conclusion on this matter^30,47^. If SL is an unconscious effect caused by the team setting, then it may be necessary to raise awareness to counteract it. On the other hand, if it is a conscious decision, then it appears to be an active choice or strategy for accomplishing the task. In this case, we need to learn why this choice or strategy was chosen and, if necessary, adjust the way redundancy is implemented or encourage individuals by certain measures to maintain their efforts. The comparison of objective and subjective SL measures can therefore help to better understand SL effects and is essential for determining suitable countermeasures.

## Experiment 1: blinded double inspection

The first experiment investigated the effects of sequential human redundancy on participants’ search effort and detection performance (i.e., search outcome) in a blinded visual inspection task. In this blinded process, findings from the first team partner (R1, RedundantPosition1) were not transferred to the second team partner in the sequence (R2, RedundantPosition2). Also, team partners did not know each other and did not receive any explicit information concerning their team partners’ reliability. In this first experiment, we tested our hypothesis that human sequential redundancy leads to SL in both positions in the sequence, compared to a condition where only one individual is responsible for the task (NonR; NonRedundant). This was grounded in the lower evaluation potential of individual contributions in the team setting^27,28,32–34^ and the fact that the individual contributions in double inspection are redundant^35^. Regarding the SL effects at the different inspection positions (R1 and R2), we did not expect any large differences as key factors influencing SL (degree of identifiability, reliability information of the team partner) were the same in both positions^30^. Social-loafing effects should be reflected in a reduced effort in the visual inspection compared to NonR participants, which may manifest either in the inspection area or the inspection duration. If a reduced search effort occurs in the redundant groups, this consequently may also lead to a lower failure detection and false alarm rate. Despite possible negative SL effects at the individual level, we hypothesized that the integrated team performance of two individuals would still exceed that of individuals working alone. The hypothesis was based on previous findings showing that the effects of SL tend to be rather moderate^19,30^, whereas the redundancy gains of units with intermediate reliability are typically strong^48^.

In addition, this experiment addressed two other aspects in an exploratory manner. The first one included the question of to what extent a reduction of search effort represented the result of a conscious decision. Thereto subjective and objective effort was compared. Another aspect included possible changes in task effort and task outcome across time. This aspect was addressed through the comparison of the different measures of inspection effort and inspection outcome across different blocks of trials.

## Method

### Transparency and openness

The study was preregistered at the Open Science Framework^49^ and was approved by the local ethics committee at the Department of Psychology, Technische Universität Berlin, Germany. This study is in accordance with the APA’s Ethical Principles. Participants took part voluntarily, gave their informed consent, and were debriefed after the experiment. Data, analytic code and video recordings of the experimental paradigm are available via the Open Science Framework (OSF) at https://osf.io/vnhx3/?view_only=2e53f862390b49708a5cfce8e38ea787.

### Participants

Based on a G*Power^50^ calculation, a sample size of *N* = 51 should suffice to detect large between-subject effects and medium within-subjects and interaction effects in our analyses (ANOVAs; α err prob = 0.05, 1-β err prob = 0.80). Slightly more participants were recruited (*N* = 62) out of a university participant pool and took part in the experiment. They were randomly assigned to one of three conditions. Specifically, 20 participants worked in the NonR condition, and 21 participants each worked in the R1 and R2 conditions respectively. Two participants (both from the R1 condition) did, however, not meet the pre-defined inclusion criteria as they produced false alarms in more than 20% of images that actually did not contain a letter. Thus, the final sample for the data analyses consisted of 60 participants. Of those 60 participants, 32 were female and one was diverse (did subjectively or objectively not identify with either the male or the female sex only); 33 were students. Their ages ranged from 20 to 40 (*M* = 29.95, *SD* = 5.56). Participants were compensated with €20.

### Apparatus and task

**Fig. 1:**
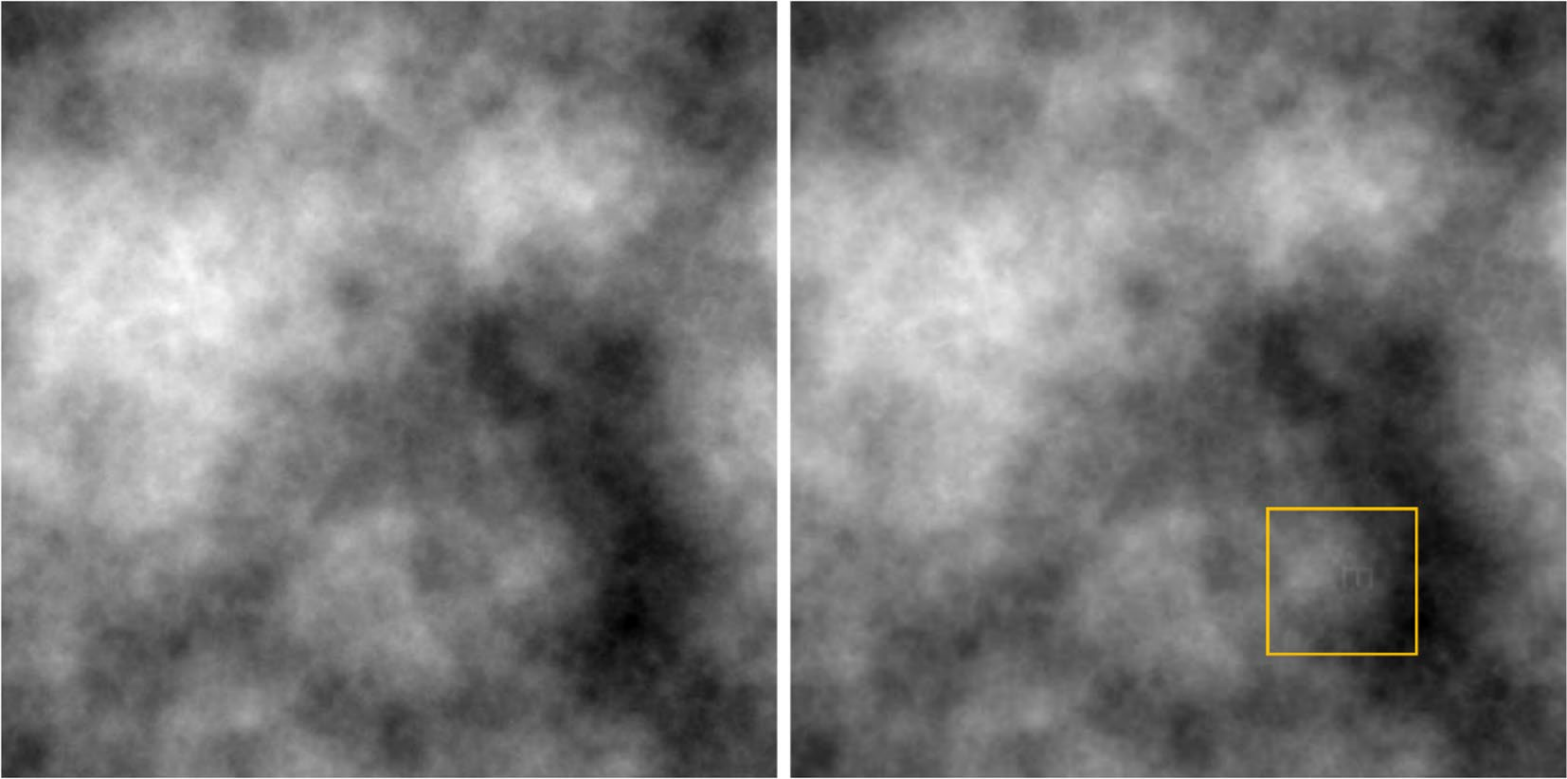
Image without the sharpening tool (left) and with the sharpening tool over the target letter E (right).

Each participant sat on a workstation consisting of a standard Windows computer, with a 24-inch screen and a 1920 × 1200 resolution, equipped with a small numeric keyboard and a standard computer mouse. Participants performed a visual inspection task that mimicked a medical X-ray examination. The task was to search simulated mammography images for critical structures (targets), which were defined as occurrences of the letter “E” embedded in the noise of the image. Critical structures had to be distinguished from similar, but non-critical structures, which were defined as occurrences of the letter “F”, analogous to distinguishing true cancer indicators from areas that look similar but are harmless. The noise had a power spectrum of 1/*f*^3^ which resembles the power spectrum of mammograms^51^ and was the same as used in previous research on visual search^52,53^. The letters were depicted from different angles by rotating them by 0, 90, 180 or 270 degrees. Images were 512 × 512 px in size and contained no, one or two letters, that were placed in randomly selected locations without overlapping each other. Note that images were initially slightly blurred so that targets were not directly visible. Only, when the computer mouse was over an image a small yellow rectangle occurred that enabled participants to gradually sharpen parts of the images within the rectangle. Therefore, targets and distractors only became detectable by moving the sharpening tool over the image surface with the mouse. The purpose of the sharpening tool was to assess search behavior and to track how much of the image participants uncovered. Figure 1 shows an example of an image, that is once blurred and once partially sharpened by the sharpening tool.

**Fig. 2:**
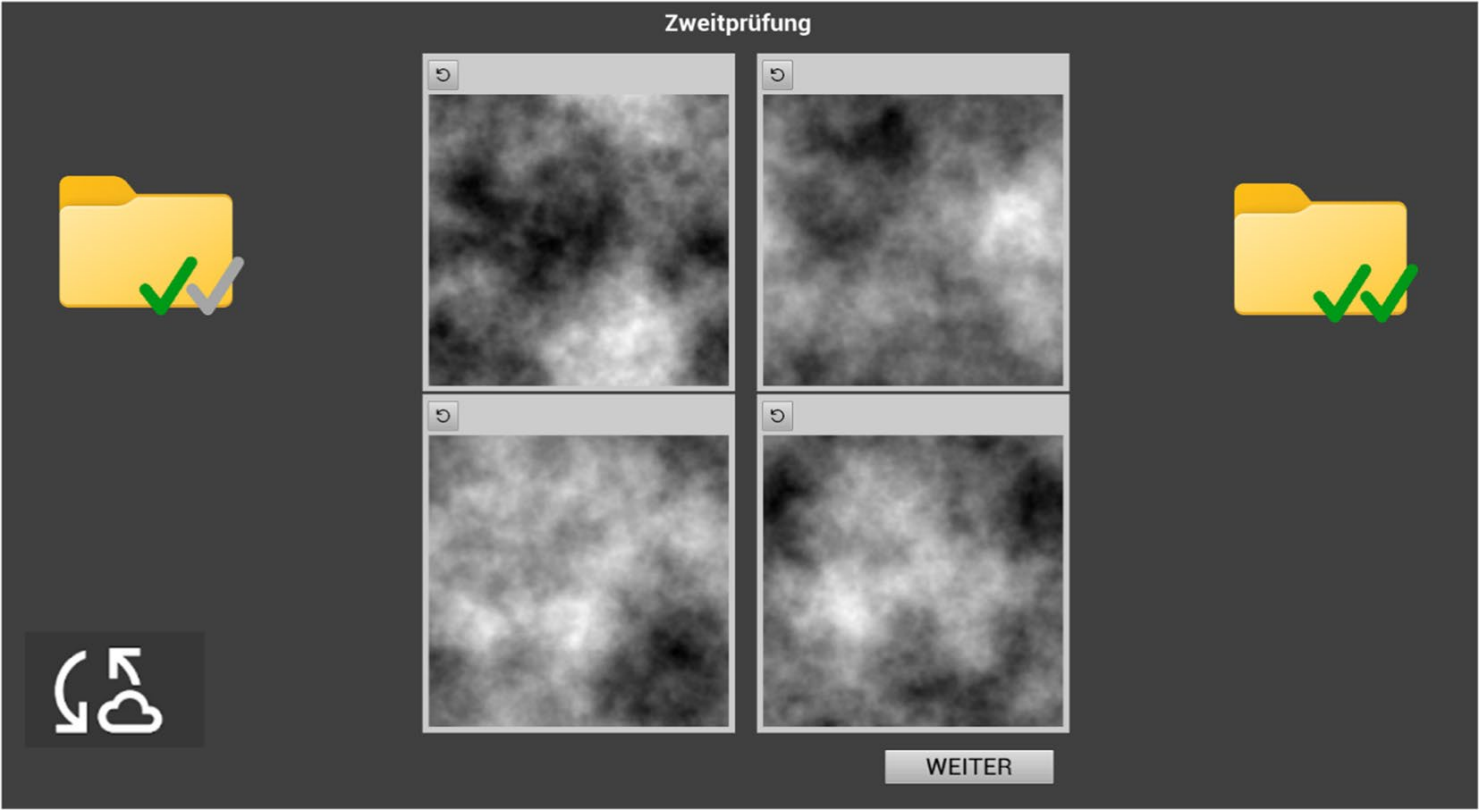
User interface as seen in the second inspection position.

**Fig. 3:**
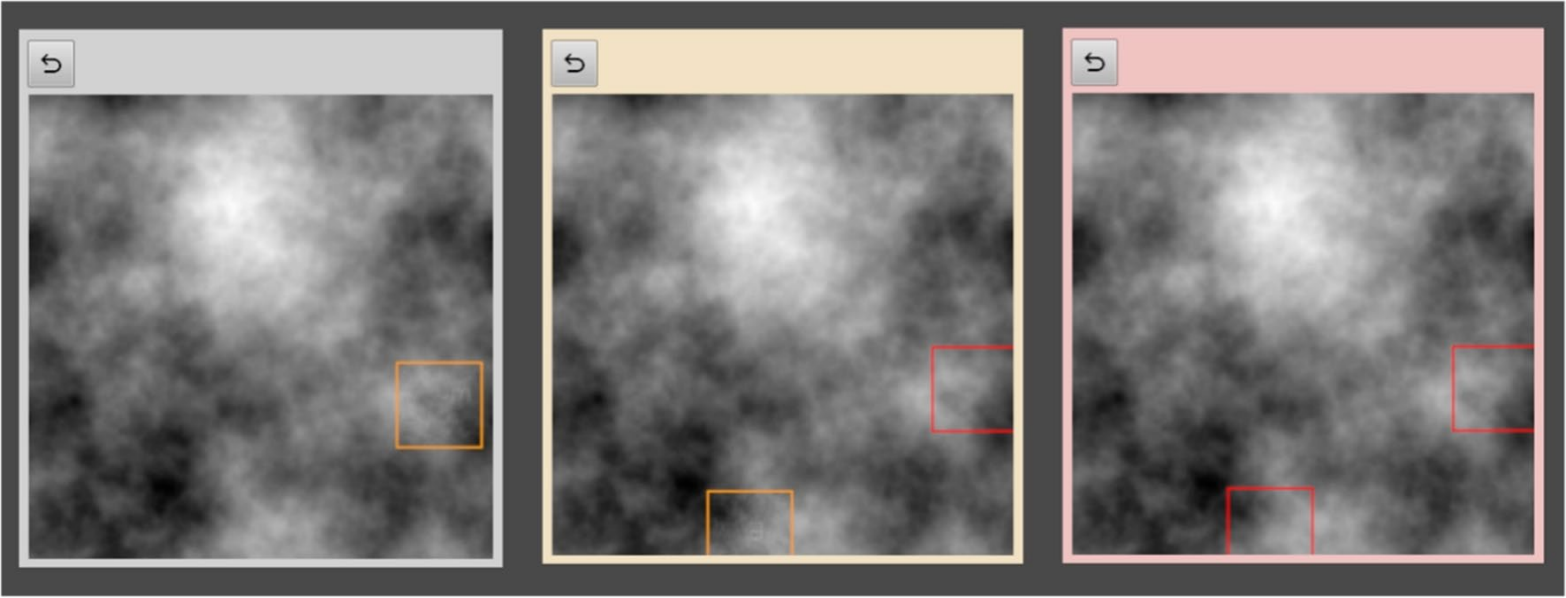
Grey: Image with sharpening tool (yellow square) revealing the target letter E. Yellow: A first target is marked with a red square, a second target is visible with the sharpening tool, and the yellow image frame indicates that the search on the four images should ideally be finished soon. Pink: Both targets are marked with a red square, the pink image frame indicates that the search takes longer than it should.

The interface of the visual inspection program is depicted in Fig. 2. In the center, four images were presented at the same time similar to radiologists who see four images per woman (each breast from different angles) when they search for breast cancer. Whenever participants saw the target “E”, they were required to left-click it to set a red-squared marking around it (see Fig. 3). If the participants made an accidental marking or changed their mind, a marking could also be taken back by clicking on an undo button (above each image on the left). On the left side of the interface, a folder was visible that contained images not yet inspected by the participant, whereas all screened images were sorted into the right folder after inspection. In the current experiment, one folder contained 40 images, and two folders were later merged into one block during data analysis.

A visual time-pressure component was integrated. The frame around the images that was initially light grey turned yellow 24 s after the images appeared and turned pink after a further twelve seconds (see Fig. 3). Participants therefore had, on average, nine seconds per image until the pink frame coloring appeared, but they could theoretically search as long as they wanted, as the search was not externally terminated.

Participants either worked alone or redundantly on the visual inspection task. A few program adaptations were made to fit these two scenarios. In the one-person setting, the heading said ‘inspection’, but in the case of the redundant task, the first person in line saw the heading ‘first inspection’, and the second saw the heading ‘second inspection’ at the top of their interface. Also, depending on whether it was the only, the first or the second inspection, the check marks on the folders on both sides were partially or completely grey (not yet inspected) or green (inspected). Further, in the team setting members needed to log in to the program by entering the IP address of their computer (which was written on their monitor) in order to be presumably connected to the team partner. Only after both team partners were logged in would the program start for both of them simultaneously. This joint login and the mutual waiting for each other aimed to make the teamwork very perceptible despite sitting visibly separated from each other. It is important to note that, in reality, the computers were not connected. To further reinforce the impression that the teams were actually working together, a connection icon occasionally flashed in the team condition. In position 1, the connection icon flashed on the right side under the folder that collected the checked images as an indication that the folders were being submitted for secondary checking. In position 2, the connection icon appeared on the left side of the interface as an indication that the team partner's folders that had been checked were arriving. While the person in the second position had to wait for the first checked folder from position 1, people in position 1 were able to start the search task immediately after logging in together. During the training, the waiting time for position 2 was set to two minutes, while the waiting time before the main experiment took about eight minutes. This delayed start of the secondary assessment was intended to reinforce the impression that both team partners were actually working on the task sequentially.

### Design

A 3 (group) × 4 (block) mixed-factorial design with repeated measurements of the second factor was used for the experiment. The first factor included three different conditions. One-third of the participants worked alone (NonR), and the remaining two-thirds of participants worked in a sequential redundancy setting in the visual inspection task, either as the first (R1) or as the second team member in line (R2). The second factor represented a within-subjects factor that was included to exploratively investigate whether the checking effort and/or possible SL effects were influenced by time-on-task. All participants saw the same 320 images in the visual inspection task. The images were singularly randomized, meaning that all participants saw the same images in the same order. For the data analysis, the 320 images were split into four sequential blocks of 80 images each.

### Dependent variables

Three sets of dependent variables were used. The first set of variables included individual search effort and search outcome measures on the individual level. To investigate the search effort, we assessed the area that was inspected via the computer mouse in percent and the time that participants invested to sharpen/scrutinize images in seconds. We chose these two variables because all participants had to find a strategy to balance speed and accuracy in completing the rather difficult task. If people reduced their effort, they could either uncover the entire area but invest little time (with the risk of uncovering targets but not seeing them) or prefer to inspect thoroughly but not the entire area of the image (with high certainty that targets uncovered will be detected). Inspecting only part of an area at high speed was also theoretically possible, but less likely. To investigate the search outcome, we assessed detected targets (hits) and false alarms. False alarms were either falsely marked distractor letter “F” or falsely marked visual noise.

The second set of variables represented search effort and search outcome measures on the team level. Team data was created by combining the data of those who assumed to be working together in a team, i.e. R1 and R2 participants. The team inspection area was defined as the image area in percent that was inspected by either of the team members. The team inspection time was the sum of both individual inspection times of team members and was relevant as with more time invested overall the likelihood of detecting a critical structure in a visual search task should increase. Team hits counted all occasions where a target was found by either of the two team members. Team false alarms were the number of false marks by either of the team members.

Finally, the third set of dependent variables comprised a set of four subjective effort ratings, which correspond to the subjective items used in previous redundancy research^20^. To investigate whether possible objective effort reductions corresponded to participants’ perceptions, they had to indicate on a seven-point rating scale how much they agreed to different effort statements. Besides the two items “I put maximum effort in the search task.” (SE1) and “I put less effort into the inspection as time went on.” (SE2), which all participants had to rate, two more specific items were presented only to participants who worked redundantly. One item asked whether the same effort would have been expended in a non-redundant setting (SE3: ”I put the same amount of effort into the inspection as if I had worked on it alone.”), and the other directly queried whether the redundant setting led to more reliance and less subjective checking in order to reduce workload (SE4: ”I occasionally relied on my team partner's performance to give me a short rest.”). Note that the R2 participants were given 40 additional images to prevent them from completing the task earlier than R1 participants (if they loaf they might be faster). However, the effort ratings of R2 participants may be slightly higher due to these 40 extra images, making it harder to detect subjective effort reductions in R2 participants.

### Procedure

Up to four participants completed the experiment simultaneously each sitting at a workstation that was separated by a partition so that participants could not see each other. Upon arrival, participants chose their workstation from those still available. When teams were tested, they were informed which of the workstations belonged to a team and were interconnected to realize the double inspection. Participants gave informed consent, completed a demographic survey, and received written instructions explaining their specific experimental condition and the inspection task at hand. Participants in the nonredundant group were told that they would be working alone. Participants in the two redundant conditions were instructed that they would perform the search in the first or second position of a two-person team and that their workstations were connected to each other to enable teamwork. They were further informed that their search results would not be visible to their team partner (blinded) and that only their integrated team performance would be evaluated. All participants were asked to find as many targets as possible, but also to keep the inspection efficient, i.e., to minimize the pink coloring of the image frames. After reading the illustrated written instructions, participants practiced the task and then completed a questionnaire that checked their understanding. After clarifying any misunderstandings or remaining questions, the data collection started.

Each participant received an identical set of *N* = 320 images. Since the base rate of the searched features in many checking tasks that apply redundancy (e.g., mammography screening, luggage screening) is very low^2,3^,targets occurred rarely in the present study. Only ten percent of the images (32 out of 320) contained either one or two targets (E). Altogether, 36 target letters and 92 distractor letters (F) were evenly distributed across four blocks of 80 images each (see Table 1). The stimulus order for the experiment was randomly generated, but the same for each participant. However, participants second in line received 40 additional images after the 320 images to ensure that even a person who was putting little effort into the task, would not have finished before the participant first in line. These 40 images were not included in the data analyses. In total, each experimental session took about an hour (*M* = 56.04 min *SD* = 13.59). At the end of each session, subjective variables were assessed, participants were debriefed and received their compensation.

**Table 1:** The frequency of images with different characteristics.

Image with	F	E	FE	EE	FF	No letter
%	15	5	3,75	1,25	5	70
Total/per block	48/12	16/4	12/3	4/1	16/4	224/56

### Statistical analysis

In the case of outliers in our data that deviated from the median by more than three times the interquartile range from their specific group, they were winsorized, i.e., their value was replaced by the nearest not suspect value^54^. To analyze our data, we conducted analyses of variance (ANOVAs) and post-hoc comparisons. Alpha was set at 0.05, and Holm-Bonferroni corrections were applied for post-hoc t-tests. If the sphericity assumption was violated, we used Greenhouse–Geisser corrections. We report effect sizes of ANOVAs using η_p_^2^ (partial eta-squared) with values of 0.01, 0.06, and 0.14 being interpreted as small, medium, and large effects respectively^55^. In the event of strong violations of the normal distribution, we used non-parametric tests and reported the Pearson's correlation coefficient r as an effect size with values 0.1, 0.3, and 0.6 being interpreted as small, medium, and large effects^55^.

## Results

### Inspection area

**Fig. 4:**
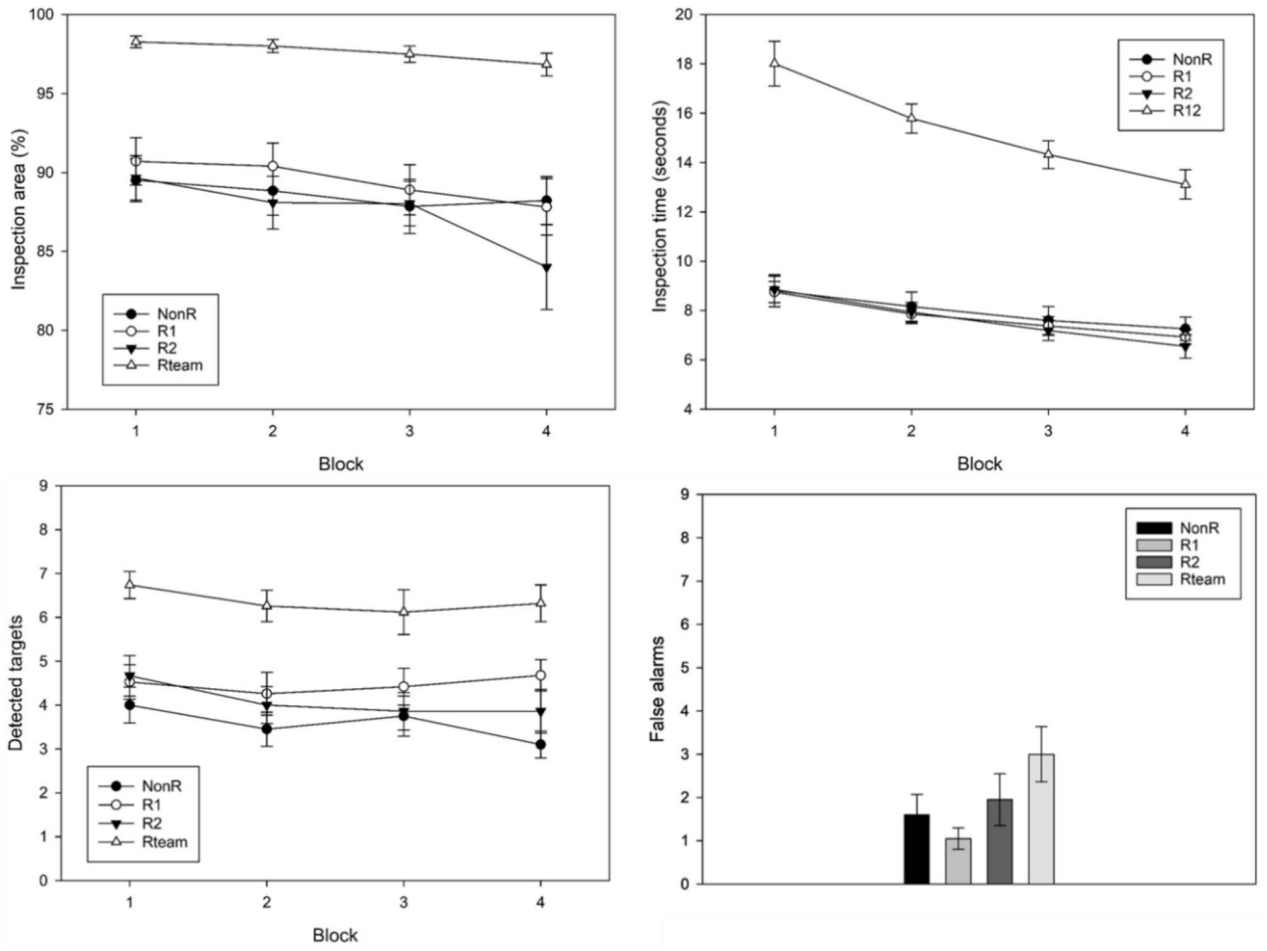
Results of blinded double inspection. Top left: average inspection area per group across the 4 blocks with standard errors. Top right: average inspection time per group across the 4 blocks with standard errors. Bottom left: average number of detected targets per group across the 4 blocks with standard errors. Bottom right: average number of all false alarms per group across the experiment with standard errors.

A few extreme values were winsorized (1.0% of all data points). The means and standard errors of the inspected image area are shown in Fig. 4 (top left). A 3(group) × 4(block) mixed ANOVA was performed with the three individual groups NonR, R1 and R2, and the four successive blocks of 80 images each. The analysis revealed a significant main effect of block, *F*(1.3, 74.09) = 8.19, *p* = 0.002, η_p_^2^ = 0.13, as slightly less image area was inspected with ongoing time. No main effect of group,* F*(2, 56) = 0.13, *p* = 0.876, η_p_^2^ = 0.005 (*M*_*NonR*_ = 88.61, *M*_*R1*_ = 89.46, *M*_*R2*_ = 87.45), nor an interaction effect, *F*(1.32, 74.09) = 1.20, *p* = 0.312, η_p_^2^ = 0.04, was found.

A second 2(group) × 4(block) mixed ANOVA was performed to compare the NonR with the Rteam group. Besides a main effect of block, *F*(1.91, 68.57) = 3.82, *p* = 0.028, η_p_^2^ = 0.10, the expected main effect of group was found, *F*(1, 36) = 31.88, *p* < 0.001, η_p_^2^ = 0.47, as teams of two people jointly uncovered more area than a single person (*M*_*Rteam*_ = 97.65, *M*_*NonR*_ = 88.61). The interaction was not significant,* F*(1.91, 68.57) = 0.812, *p* = 0.443, η_p_^2^ = 0.02.

### Inspection time

A few extreme values were winsorized (2.5% of all data points). The means and standard errors of the invested inspection time per image are shown in Fig. 4 (top right). The graphs for R1, R2 and the NonR group are very similar. Participants on average searched for *M* = 8.80 s in block 1 and got faster over time. In block 4 they invested only *M* = 6.91 s per image. Thus, the average search time of all participants in all blocks was below nine seconds, indicating that participants were efficient and avoided the pink coloring of the image frame after 36 s. A 3(group) × 4(block) mixed ANOVA revealed a significant main effect of block, *F*(1.75, 92.55) = 46.46, *p* < 0.001, η_p_^2^ = 0.47. Neither a main effect of group, *F*(2, 53) = 0.96, *p* = 0.390, η_p_^2^ = 0.04, nor an interaction effect was found, *F*(3.49, 92.55) = 0.18, *p* = 0.935, η_p_^2^ = 0.01.

A second 2(group) × 4(block) mixed ANOVA with only the NonR and Rteam group (*M*_*NonR*_ = 7.95, *M*_*Rteam*_ = 15.31) showed a main effect of block, *F*(1.52, 53.06) = 39.26, *p* < 0.001, η_p_^2^ = 0.53, and a large main effect of group, *F*(1, 35) = 87.14, *p* < 0.001, η_p_^2^ = 0.71. The interaction effect was significant, too,* F*(1.52, 53.06) = 6.44, *p* = 0.006, η_p_^2^ = 0.16. A look at Fig. 4 (top right) reveals a steeper decline for Rteams than for the NonR group.

### Detected targets

A 3(group) × 4(block) mixed ANOVA revealed no significant main effect of block, *F*(3, 171) = 1.29, *p* = 0.278, η_p_^2^ = 0.02. The main effect of group was not significant either, *F*(2, 57) = 2.03, *p* = 0.141, η_p_^2^ = 0.07 (*M*_*NonR*_ = 3.58, *M*_*R1*_ = 4.47, *M*_*R2*_ = 4.10). No significant interaction effect was found, *F*(6, 171) = 0.59, *p* = 0.738, η_p_^2^ = 0.02.

The 2(group) × 4(block) mixed ANOVA comparing the NonR with the Rteam group did not find a main effect of block, *F*(3, 111) = 1.35, *p* = 0.263, η_p_^2^ = 0.04. However, a large main effect of group was found, *F*(1, 37) = 49 0.23, *p* < 0.001, η_p_^2^ = 0.571, as teams detected more targets than the participants from the NonR group that worked alone (*M*_*Rteam*_ = 6.38, *M*_*NonR*_ = 3.58). No interaction effect was found, *F*(3, 111) = 0.411, *p* = 0.746, η_p_^2^ = 0.01.

### False alarms

Only very few false alarms were set off. Figure 4 (bottom right) shows the means of the winsorized (3.9% of data points) number of false alarms per experiment. False alarms can be either wrong marks on distractors or wrong marks on random visual noise. The latter case was much more frequent (74% of false alarms). Almost no letter Fs were mistaken for the target letter E. Due to the low number of false alarms and their nonnormal distribution, Mann–Whitney-U tests were performed to compare the groups. Participants from the NonR, R1, and R2 groups did not differ significantly, all *p* > 0.346.

However, the teams on average set off *M*_*Rteam*_ = 3.00 false alarms in 320 images (*SE* = 0.64) and participants from the NonR group only *M*_*NonR*_ = 1.60 (*SE* = 0.47). This difference was significant, *U* = 127.00*, Z* = -1.81, *Bonferroni-Holm p* = 0.035, *r* = 0.29.

### Subjective ratings

Mean ratings of subjective ratings are presented in Table 2. Kruskal–Wallis-H was used and found no differences between experimental conditions for all subjective measures, all *p* > 0.553. Participants in all groups reported that they had put a lot of effort into the search task. They reported that their effort did not decrease with time on task, and they claimed to have worked as hard as if they had been working alone.

**Table 2:** Means (and standard errors) of subjective effort ratings on a seven-point Likert scale (1 = fully disagree; 7 = fully agree) in experiment 1 (blinded).

	NonR	R1	R2
SE1: I put maximum effort into the search task	6.05 (0.15)	5.79 (0.34)	5.90 (0.43)
SE2: I put less effort into the inspection as time went on	3.25 (0.45)	3.21 (0.44)	3.45 (0.38)
SE3: I put the same amount of effort into the inspection as if I had worked on it alone	–	5.89 (0.45)	6.15 (0.51)
SE4: I occasionally relied on my team partner's performance to give me a short rest	–	1.68 (0.27)	1.60 (0.22)

## Discussion

This experiment tested whether SL occurs when a visual inspection is performed by two humans that work sequentially redundant and when no information is passed from the first to the second team member. SL was theoretically possible in this setting due to the lower identifiability of individual contributions that is related to a lower evaluation potential^27,28,32–34^, and the risk of a loss of motivation caused by the fact that participants working in a team did the exact same task and did not contribute uniquely^35^. We assumed that both the first person and the second person in the redundant work setting might show signs of SL to a comparable degree, e.g., show a reduced image inspection time, a reduced image inspection area, fewer detected targets, and fewer false alarms compared to people working alone on the task. Nevertheless, we expected that despite possible SL the combined team inspection time, inspection area and target-detection performance should surpass that of a person being solely responsible for the visual inspection task. However, on the downside, the teams were assumed to set off more false alarms than people working alone on the inspection^13,41^.

Looking at the results we need to discard all our SL hypotheses. The results of the experiments are not indicative of a reduced task effort or task outcome in both groups that worked in the redundancy setting (groups R1 and R2). The subjective ratings also showed no evidence of SL, as all groups stated to have worked hard. Since no objective and subjective effort reductions were found, no conclusions can be drawn about whether SL was performed consciously or not. Overall, our results are in line with the sequential redundancy research obtained in a multitasking setting^20^, which did not find SL when participants double-checked the quality of chemicals in a blinded sequential-redundancy setting.

Due to the lack of SL, the expected redundancy effect became very evident. The cumulated search time of the two-person teams was more or less twice as much as that of the NonR participants. And while the proportion of the image searched was already very large for all individual groups (between 84 and 91%), teams still significantly improved the inspected image area to 98%, which was about ten percent higher than that of the NonR participants. Also, for the number of targets detected per block, a clear team superiority was found. While teams detected *M*_*Rteam*_ = 6.38 defects on average (70,88%), only *M*_*NonR*_ = 3.58 defects (39,78%) were detected per block in the NonR condition. Theoretically, two individuals of the NonR group combined should detect 64,93% of the targets ([1−(5.42/9)^2^] x 100). The actual team detection performance was thus even slightly better than one would expect from two independent redundant inspectors (2 × NonR). However, despite this positive result, the team target detection was still far away from the optimum of nine detected targets per block. Remember that participants were instructed to find all targets and could search as long as they wanted. However, they were also told to keep the search efficient by avoiding the image frame to stay pink for a long time. The average image inspection time shows that participants paid attention to the time-pressure element. At the same time, participants inspected a high image area, yet many targets remained undetected. In principle, all targets were detectable (each target was detected by multiple participants) but it seems that the low prevalence of targets and the search speed paced by the time pressure manipulation made it rather difficult to detect all targets.

In conclusion, similar to the previous findings within a multitask setting^20^, we did not find indices of SL in a realistic visual inspection using a blinded sequential-human-redundancy work setting. It seems as if the pure knowledge of the task being done twice does not lead to a great reduction of task effort and task outcome that can endanger the gains of human redundancy if the task completion remains more or less independent (i.e., no direct interaction and communication during task, no information transmission, working with a stranger) and both participants have an equally low task expertise. However, in our multitasking study using the nonblinded sequential redundancy^20^, where marks were transferred from the first to the second team member, a large SL effect occurred in the second inspection position. Thus, experiment 2 was conducted to investigate whether work settings that use nonblinded sequential human redundancy are also prone to SL when no parallel tasks need to be performed.

## Experiment 2: nonblinded double inspection

This experiment investigated the effects of sequential human redundancy on participants’ search effort and search outcome in the same image-inspection task as experiment 1, but this time under nonblinded conditions, in which decisions (in the form of set marks) made by the first inspector were forwarded to the second inspector. Note that the second inspectors received not the actual decisions made during the first inspection, but instead saw marks set by a scripted highly reliable first inspector. This was done because social loafing is more likely when working with team partners that perform well^30^. We therefore tried to create a setting that would increase the chances of participants second in line to start loafing similar to past research using a multitasking setting^20^. Remember that past research could not find social loafing when working with a moderately reliable team partner^21,26^ and that a low performing partner can even lead to social compensation behavior (i.e., an increase in effort)^18,26^. We hypothesized that team members first in line would not engage in SL, as their individual contribution could be assessed by team members second in line^20,27,28,32–34^. However, we hypothesized that inspectors second in line, realizing that their preceding team partner already worked highly reliably, would be more prone to SL, especially as time on task and trust in the team partner would rise^20,26,30^. Remember that the fact that second inspectors saw local marks on specific image areas containing a potential target, offers even greater potential to reduce inspection effort in the second position compared to a global alert that only informs that the picture contains somewhere a potential target^20^. Theoretically, participants seeing local marks set by their team partner, might verify these areas only or scan through unmarked areas much faster. We hypothesized that SL should be reflected in a reduced inspection time and inspection area, and eventually even in a lower detection of targets. On the positive side, there may also be fewer false alarms. However, since substantial SL effects were only hypothesized to arise in the second position, we still assumed that, as in experiment 1, the overall team effort (inspection time and area) and team task outcome (targets detected) would be somewhat better than that of people working alone, thus reflecting a perhaps diminished but nevertheless visible advantage of the redundant work setting for all objective measures but false alarms. It was again expected that false alarms would be higher for teams than for people inspecting alone^13,41^.

## Method

### Transparency and openness

The study was preregistered at the Open Science Framework^56^ and was approved by the local ethics committee at the Department of Psychology, Technische Universität Berlin, Germany. This study is in accordance with the APA’s Ethical Principles. Participants took part voluntarily, gave their informed consent, and were debriefed after the experiment. Data, analytic code and video recordings of the experimental paradigm are available via the Open Science Framework (OSF) at https://osf.io/vnhx3/?view_only=2e53f862390b49708a5cfce8e38ea787.

### Participants

A total of *N* = 62 participants recruited out of a university participant pool took part in the experiment and were randomly assigned to the three groups. Specifically, 22 participants worked in the NonR group, and 20 participants each worked in the R group. However, five participants (NonR: 3, R1: 1, R2: 1) did not meet the pre-defined inclusion criteria but produced false alarms in more than 20% of images that did not contain a letter. Thus, the final sample included in the data analyses consisted of 57 participants. Of those 57 participants, 20 were female and two were diverse (did subjectively or objectively not identify with either the male or the female sex only); 38 were students. Their ages ranged from 20 to 40 (*M* = 26.58, *SD* = 4.66). Participants were compensated with €20.

### Apparatus and task

**Fig. 5:**
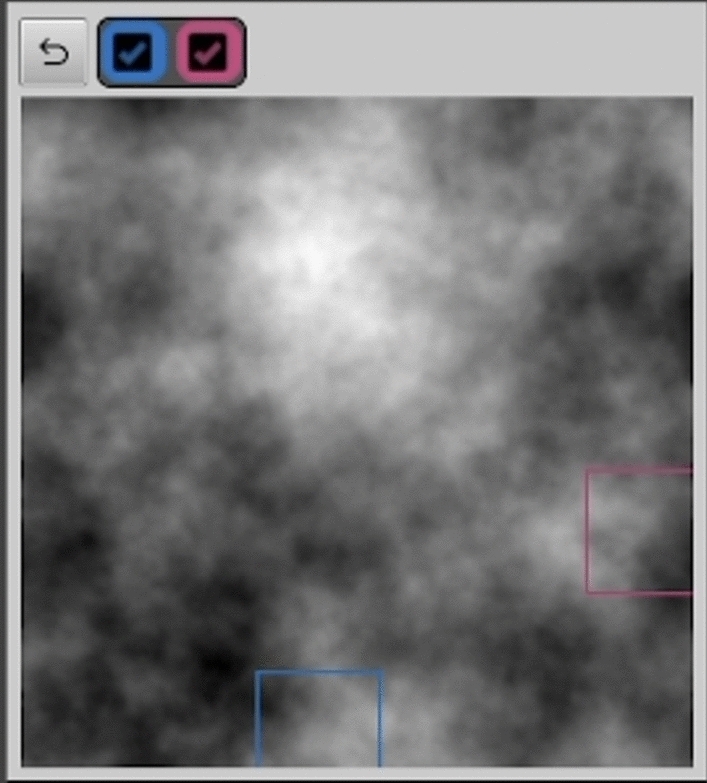
Target marks set during the first inspection are displayed to R2 participants. These marks could be deselected (by clicking on the check symbol) in the menu above the image in case participants did not agree with the marks.

The same experimental setup was used as in experiment 1. Participants performed a visual inspection task searching for the letter 'E'. However, in this experiment, a nonblinded process was implemented. Therefore, participants in position 1 were told that their inspection results would be transferred to the person in position 2, and participants in position 2 saw marks presumably set by a their team partner in position 1. While the interface for the NonR and R1 groups was the same as in experiment 1, the interface was slightly different for the R2 group. The difference was that the participants second in line saw marks on the images from their putative teammates set around the image area that contained the potential target (Fig. 5). These marks could be deselected in the menu above the image in case participants did not agree with the marks (Fig. 5). Of course, additional marks could be set. It is important to note that the marks that were displayed to participants in position 2, did not correspond to the real marks of the participants working first, but instead were scripted so that all participants in position 2 worked with a highly reliable team partner.

### Design

The design resembles that of experiment 1. Again, a 3 (group: NonR, R1, R2) × 4 (block) mixed-factorial design with repeated measurements of the second factor was used for the experiment. All participants saw the same 320 images in the visual inspection task. For the data analysis, this number was again split into four blocks of 80 images each. The R2 group saw correct target marks during the first three blocks and no target marks in block 4, opening up the opportunity to miss 9 targets in block 4. This setting (i.e., conservative criterion shift in the scripted R1) was chosen to mimic situations where a generally highly reliable coworker becomes temporarily tired or distracted. Marks of distractor letters or unsuspicious noise areas were not scripted and did not occur in the experiment.

### Dependent variables

Dependent measures were again defined as search effort and search outcome measures on the individual level and on the team level. Specifically, we looked at inspection area, inspection time, detected targets and false alarms. However, compared to experiment 1, only in block 4 participants from all groups saw no target marks. Therefore, only the detection rate of the unmarked *N* = 9 targets in block 4 was compared across all groups. The same ratings as in experiment 1 were used to assess subjective effort.

### Procedure

The procedure followed that of experiment 1 with the important exception of the simulated transmissions of information from R1 to the R2 position (see description under ‘Apparatus and task’).

### Statistical analysis

The statistical analysis was similar to that of experiment 1 but differed in some parts due to the transmission of target marks from position 1 to position 2. Specifically, R2 participants saw correct target marks in block 1–3 but no target marks in the failure block 4. Therefore, for targets detected, only block 4 was compared between all groups. The subjective effort ratings, that were queried from team members only, were compared using one-sided Kruskal–Wallis-H test since a priori directed hypotheses existed for effort in both inspection positions (R1: no SL, R2: SL).

## Results

### Inspection area

**Fig. 6:**
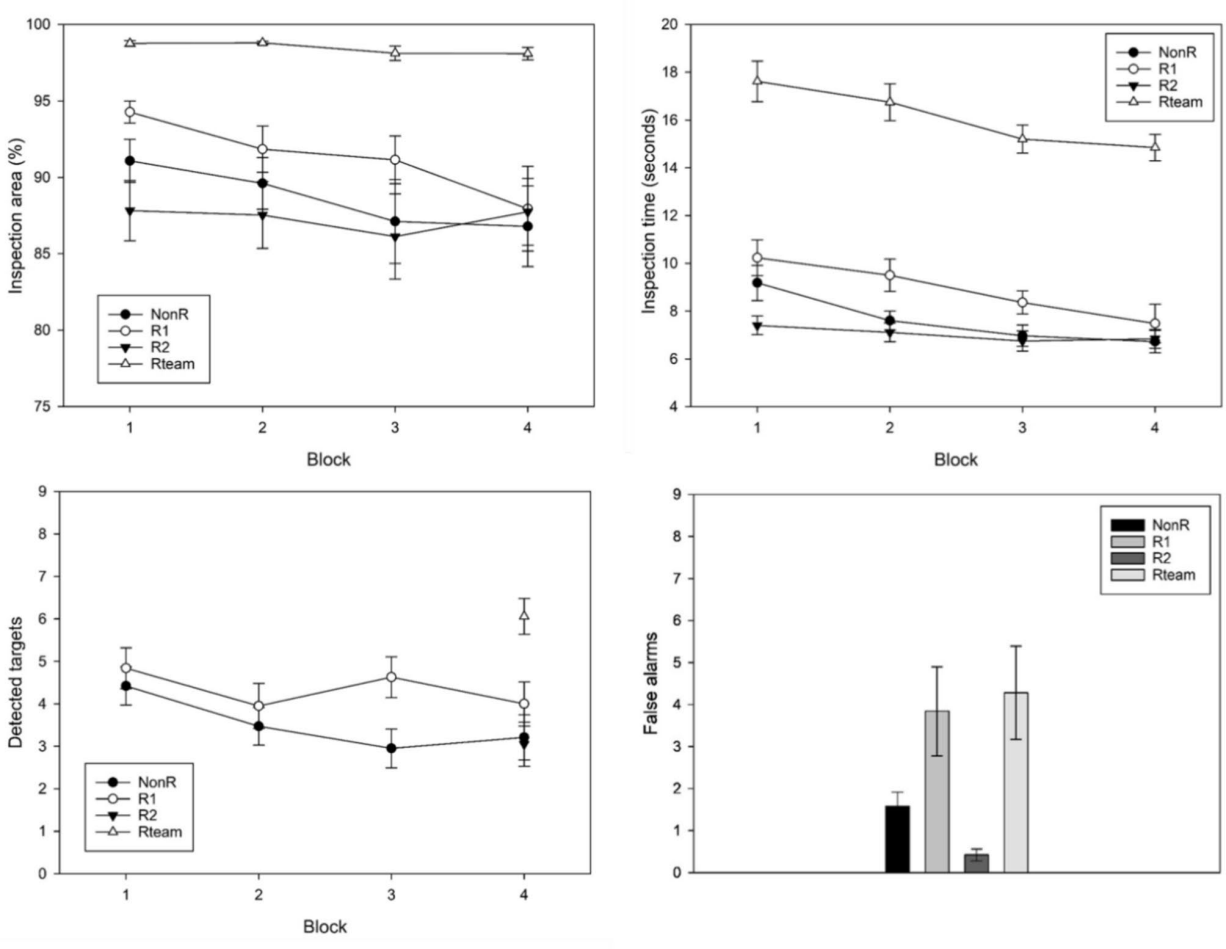
Results of nonblinded double inspection. Top left: average inspection area per group across the 4 blocks with standard errors. Top right: average inspection time per group across the 4 blocks with standard errors. Bottom left: average number of detected targets per group across the 4 blocks with standard errors. Bottom right: average number of all false alarms per group across the experiment with standard errors.

A few extreme values were winsorized (3.0% of data points). The means and standard errors of the image area inspected per group and across the blocks are shown in Fig. 6 (top left). Averaged across the blocks, teams inspected* M*_*Rteam*_ = 98.45% of the image, R1 participants *M*_*R1*_ = 91.30%, NonR participants *M*_*NonR*_ = 88.65%, and R2 participants *M*_*R2*_ = 87.30%. A 3(group) × 4(block) mixed ANOVA was performed and revealed a significant main effect of block, *F*(1.85, 99.81) = 10.04,* p* < 0.001, η_p_^2^ = 0.16, as inspection area decreased with time on task. No main effect of group was found, *F*(2, 54) = 1.06, *p* = 0.353, η_p_^2^ = 0.04. However, the interaction effect was significant, *F*(3.70, 99.81) = 2.60, *p* = 0.046, η_p_^2^ = 0.09. A look at Fig. 6 (top left) reveals a declining inspection area in the R1 and NonR groups versus no decline or even a small increase of inspection area in the last block in the R2 group. This late effort increase in the R2 group is probably caused by the misses of the scripted R1.

A further 2(group) × 4(block) mixed ANOVA was performed to compare the NonR with the Rteam. Here beside a main effect of block, *F*(1.17, 41.07) = 6.86, *p* = 0.009, η_p_^2^ = 0.16, the expected main effect of group was found, *F*(1, 35) = 21.01, *p* < 0.001, η_p_^2^ = 0.38, as teams searched about ten percent more image area than the NonR group. The interaction was not significant, *F*(1.17, 41.07) = 3.34, *p* = 0.069, η_p_^2^ = 0.09.

### Inspection time

The means and standard errors of image inspection time per group are shown in Fig. 6 (top right). One outlier was winsorized (0.3% of data points). Averaged over the blocks two-person teams spent *M*_*Rteam*_ = 16.04 s inspecting an image, the R1 group spent *M*_*R1*_ = 8.89 s, the NonR group spent *M*_*NonR*_ = 7.62 s and the R2 group spent *M*_*R2*_ = 7.02 s per image. The 3(group) × 4(block) mixed ANOVA revealed a significant main effect of block, *F*(1.46, 78.63) = 19.38, *p* < 0.001, η_p_^2^ = 0.26, as generally less time per image was invested with increasing time on task. The main effect of group was significant as well, *F*(2, 54) = 5.21, *p* = 0.009, η_p_^2^ = 0.16. Post-hoc t-test revealed that R1 participants invested significantly more time in the inspection than R2 participants, *Bonferroni-Holm p* = 0.007. The difference between R1 and the NonR group just missed the conventional level of significance, *Bonferroni-Holm p* = 0.072. The interaction effect was significant, *F*(2.91, 78.63) = 2.77, *p* = 0.049, η_p_^2^ = 0.09. A look at the graphs (Fig. 6 top right) shows a rather constant inspection time across the blocks in the R2 group, while there is a decrease across the blocks in the NonR and R1 groups.

The second 2(group) × 4(block) mixed ANOVA to compare the NonR with the Rteam group found a main effect of block, *F*(1.40, 49.06) = 16.92, *p* < 0.001, η_p_^2^ = 0.33, and a main effect of group, *F*(1, 35) = 142.85, *p* < 0.001, η_p_^2^ = 0.80. The interaction was not significant, *F*(1.40, 49.06) = 0.82, *p* = 0.407, η_p_^2^ = 0.02.

### Detected targets

Figure 6 (bottom left) shows the detected targets per block for each group. No data is provided for blocks 1–3 for the R2 and Rteam groups. This is because R2 participants could only detect targets (that were not previously detected by the scripted R1 team partner) in block 4. A 2(group) × 4(block) mixed ANOVA was performed to compare the NonR and R1 groups across all 4 blocks (both groups don’t see marks in all four blocks). The ANOVA revealed a main effect of block, *F*(3, 108) = 4.17, *p* = 0.008, η_p_^2^ = 0.10, but no main effect of group, *F*(1, 36) = 2.24, *p* = 0.143, η_p_^2^ = 0.06 (*M*_*R1*_ = 4.36, *M*_*NonR*_ = 3.51), nor an interaction effect, *F*(3, 108) = 1.60, *p* = 0.193, η_p_^2^ = 0.04.

In block 4, all groups could detect a total of nine targets and a team score could be calculated. A one-way ANOVA comparing the R1, R2 and NonR groups (*M*_*NonR*_ = 3.21, *M*_*R1*_ = 4.00, *M*_*R2*_ = 3.05, *M*_*Rteam*_ = 6.06), found no significant difference, *F*(2, 54) = 0.94, *p* = 0.398, η_p_^2^ = 0.03. However, as expected, the second analysis comparing the Rteam score with the NonR group of detected targets in block 4 found a significant advantage of the teams, *F*(1, 35) = 17.45, *p* < 0.001, η_p_^2^ = 0.33.

### False alarms

Mean false alarms per group were winsorized (5.3% of data points) and are depicted in Fig. 6 (bottom right). Across the experiment, Rteams committed *M*_*Rteam*_ = 4.28 false alarms on average, the R1 group* M*_*R1*_ = 3.84, the NonR group *M*_*NonR*_ = 1.58, and the R2 group only *M*_*R2*_ = 0.42 false alarms. Overall false alarms occurred rarely and were not normally distributed. Of these false alarms, 31% were marked distractor letters. The Mann–Whitney-U test showed that the R2 group made significantly fewer false alarms than group R1, *U* = 78.50*, Z* = -3.15, *Bonferroni-Holm p* = 0.004, *r* = -0.51, and group NonR, *U* = 99.00, *Z* = -2.55, *Bonferroni-Holm p* = 0.015, *r* = -0.41. False alarms of group NonR and R1 did not differ significantly (*p* = 0.221). Comparing the Rteam with the NonR group revealed a statistically significant difference, *U* = 106.00*, Z* = -2.01, *Bonferroni-Holm p* = 0.044, *r* = -0.33.

### Subjective ratings

Mean subjective ratings are presented in Table 3. Kruskal–Wallis-H was computed and found no differences between the groups. Participants in all groups rated their effort to be high and stated that they did not reduce effort with time on task (both *p* > 0.386). However, SE3 (“I put the same amount of effort into the inspection as if I had worked on it alone.”) was very close to significance, H(2) = 2.67, *p* = 0.051. The ratings of S4 were not significant, H(2) = 1.04, *p* = 0.154. Remember that the subjective effort ratings of the R2 participants may have been slightly higher due to the 40 extra images.

**Table 3:** Means (and standard errors) of subjective ratings on a seven-point Likert scale (1 = fully disagree; 7 = fully agree) in experiment 2 (nonblinded).

	NonR	R1	R2
SE1: I put maximum effort into the search task	5.89 (0.20)	5.89 (0.29)	5.89 (0.31)
SE2: I put less effort into the inspection as time went on	3.21 (0.33)	2.74 (0.27)	3.47 (0.40)
SE3: I put the same amount of effort into the inspection as if I had worked on it alone. (*)	–	6.12 (0.41)	5.18 (0.49)
SE4: I occasionally relied on my team partner's performance to give me a short rest	–	1.94 (0.30)	2.71 (0.47)

NonR = nonredundant; R1 = redundant position1; R2 = redundant position2; SE = subjective effort. * *p* ≤ .05, (*) *p* = .051.

## Discussion

This experiment investigated the effects of sequential human redundancy on task effort and task outcome in a nonblinded image-inspection task in which mostly correct decisions (in the form of set marks) made by the first inspector were forwarded to the second inspector. We assumed that participants first in line would not exhibit social loafing, but that participants second in line would. Despite some SL in position 2, we believed that teams would perform together better than participants working alone.

Let us start with position R1. One important impact of a nonblinded sequential-redundancy setting is the transmission of findings of the inspector in position R1 to position R2. Thus, the performance of R1 can directly be evaluated and assessed by R2. According to SL theory, the recognition of an evaluation potential is expected to reduce SL^27,28,32–34^. In accordance with our hypothesis, no effort reduction occurred in this position. In all objective measures, R1 participants were descriptively even slightly above the NonR group, with the largest difference in the inspection time (*p* = 0.72). Although it just missed the conventional level of significance, this difference indicates, that the evaluation potential might not only able to prevent SL but may even make people invest more effort compared to when they work alone on the task.

With regard to position R2, we had hypothesized SL, especially with time on task and the ongoing experience that R2 participants could not add much to the group outcome due to a highly reliable first inspector^20,26,30^. No significant differences between NonR and R2 participants were found for inspection time, inspection area and target detection, and R2 participants effort did also not decrease over time. However, false alarms were as expected significantly lower in the R2 group compared to the NonR group. Note, that despite no significant differences between NonR and R2 participants, R2 participants descriptively invested less time, uncovered a smaller proportion of the image, and detected a lower number of targets.

Comparing R1 and R2 participants, a significant difference was found in search time and false alarms. Thus, it is very clear that nonblinded sequential redundancy led to different effort and decision effects in the two inspection positions, once to an increase (R1) and once to a decrease (R2) compared to the NonR group (although without a significant difference to NonR). The differences between R1 and R2 participants in objective effort were also descriptively visible in one of the two subjective effort ratings (*p* = 0.051). This indicates that these effort and decision effects were not unconscious effects of group work that are difficult to influence, but rather conscious decisions, strategy or changed interpretation of the task (e.g., a quick-check mentality). It would be interesting to learn the ratio behind it to be able to antagonize effort reductions by taking appropriate countermeasures, such as providing feedback^17^ or emphasizing individual accountability for the task outcome^19^.

In our previous nonblinded multitasking study, the effort of R2 inspectors dropped considerably^20^, whereas this experiment found no significant reduction of effort in the R2 group but only a significantly lower false alarm rate. It might be that, the higher workload in the multitasking setting, had intensified participants’ tendency to put less effort into the task in the second position, similar to the tendency to offload tasks under high workload to a technological partner^45^. To further clarify the moderating effect of workload on SL for both inspection positions, future research should compare the same task either with or without parallel tasks, since the multitasking studies and the current experiments presented here differed in more than one aspect.

With regard to team performance, we hypothesized that the combined performance should be superior to that of individuals working alone on the inspection. In support of this, we found that the teams significantly outperformed the solo inspectors in terms of inspection area, inspection time, and detected targets. Rteams even detected more than one would theoretically expect of two independently working inspectors in block 4 (*M*_2 x NonR_ = 62.79%, *M*_Rteam_ = 67.33%). Therefore, a clear benefit of redundancy was found in this experiment. However, although false alarms occurred very rarely, teams as expected made more false alarms than the NonR group.

Compared to experiment 1, team performance was numerically almost identical. Teams in experiment 1 inspected *M*_*Exp1*_ = 97.65% of the image area and teams in experiment 2 *M*_*Exp2*_ = 98.64%. The averaged inspection time in experiment 1 was *M*_*Exp1*_ = 15.31 s for teams and that of experiment 2 *M*_*Exp2*_ = 16.04 s. In total, teams made *M*_*Exp1*_ = 3.00 false alarms in experiment 1, compared to *M*_*Exp2*_ = 4.28 in experiment 2. The target detection in block 4 was *M*_*Exp1*_ = 70.18% in experiment 1 and *M*_*Exp2*_ = 67.28% in experiment 2. Looking at these similar numbers, it seems as if no obvious superiority of either the nonblinded or the blinded approach appeared in our experiments.

## General discussion

Human redundancy is often used in safety–critical domains to help protect against errors. For example, mammograms are read by two radiologists, or the dose of a drug is calculated by two physicians who perform the task redundantly one after the other. However, the expected reliability gain may be compromised by social loafing (SL), i.e., a reduction of individual effort caused by the team setting. A set of studies performed in a multitasking setting found SL in a sequential-redundancy setting^20^. These studies suggested that it makes a difference whether the inspection results of the first person are transferred to the second person in line or not (nonblinded vs. blinded). Specifically, these studies found that participants working at the second position reduced their effort (compared to participants working alone) when a nonblinded process with a highly reliable first inspector was used but was absent in the blinded process. However, in both studies, the team performance was still superior to solo performance.

With the current studies, we tested whether SL occurred when participants performed a visual inspection of simulated mammographic images using either the blinded or nonblinded human redundancy approach, and without the need to address to parallel tasks. In the blinded double inspection, no reduction of effort or a reduced detection of targets occurred in both inspection positions. These results are in line with the results of the multitasking study. In the nonblinded double inspection, participants in the second position spend less time on the search compared to participants working in the first position, and made less false alarms compared to participants working first in line and participants working alone. This shows that the small structural decision to transfer inspection results from the first inspection to the second inspection had a substantial impact on the individual effort invested and decisions taken at the two inspection positions in the current single-task setting. Being first or second in line to work on a visual inspection task therefore seems to make a difference in case work results are transmitted between the redundant workers. However, compared to the multitasking study, the effort of participants working at the second position did not differ from that of participants working alone (no SL). Therefore, it appears that there is less risk of SL during redundant ‘monotasking’. The difference between the current results and the multitasking studies may be due to the absence of parallel tasks and an associated workload reduction.

Overall, in both recent studies, teams still found significantly more targets than inspectors working alone. The adage "four eyes see more than two" therefore proved true in both of our studies despite differences at the individual level. Interestingly, the team results were virtually identical in both experiments. This may be partly because the two opposite developments in the nonblinded condition (slight increase in the R1 group vs. slight decrease in the R2 group) balanced each other out. In sum, at the team level, it did not make much difference whether or not the decisions or work results of the first team member were transferred to the second team member in line.

Taken together, both forms of human redundancy appear to be particularly useful in contexts where individual performance is far below the desired level, where failures have far more serious consequences than false alarms, and where there are no competing tasks to be performed in parallel. This is often the case in high-risk areas, such as the diagnosis of life-threatening diseases, where the increased staffing and time required to perform redundant tasks can be more easily justified. However, in contexts where the costs of misses and false alarms are similarly high, redundancy may be less useful, as the gains in detection of target stimuli may be offset by the costs of increased false alarms.

Although no clearly superior approach (nonblinded or blinded) could be found in our experiments at the team level, it might be a good idea to use the nonblinded approach during the training of future screeners. If trainee screeners placed in position 2 were to repeat the inspection after a fair but not 100% reliable person, they would experience firsthand that the redundant inspection has the potential to improve target detection and that their contribution is important, while at the same time their attention would be visually guided by learning processes through the large number of correctly displayed markings.

Despite the clear redundancy gain in both current experiments, the team performance was still far away from optimal as teams detected only about two thirds of the targets. This was of course grounded in an even lower detection rate at the individual level. While participants inspected a great proportion of the images, they still did not find many targets. This might be grounded in the low prevalence of the targets and the time pressure used in our experiments. It seems as if the search speed paced by the changing frame color around the images was too high to detect all targets. Of course, time pressure is present in the health sector and at airport security checks. However, with less time pressure more targets probably would have been found in our studies. Another option would be to increase the frequency of targets by using fake targets, similar to the projection of fictional threats into X-ray images of cabin baggage^4^. Also training participants on images in which targets occur often might improve the target detection afterwards when the prevalence is low again^57^. Another approach to increase reliability could be adding a third person to the inspection team. Here it would be necessary to recheck the occurrence of SL, as having more people on the team could again increase the likelihood of SL^30^.

In summary, although human redundancy is popular in high-risk areas, the principle has not been tested for its effectiveness or its impact on the perception and behavior of people performing redundant tasks. Our work found that nonblinded and blinded sequentially redundant visual search had different effects on individual effort, but were able to increase the detection rate and the image area inspected. Our work is an important first step, but more research is needed to get a more complete picture of the effects of human redundancy.

Future research should attempt to replicate whether people in the second position in the nonblinded setting are indeed less likely to make false alarms. In our nonblinded experiment, it appeared that people working in the first position were more likely to mark nontarget areas, while people working in the second position seemed to have a more conservative response criterion and rarely made false alarms. Future research could also investigate whether workload moderates SL, explore the influence of time pressure, or test whether a third person in the sequence further improves the detection of targets. Besides this, longer working times seem appropriate to be investigated as participants in our studies decreased their effort with time to a different extent. Team partners with varying degrees of reliability and the transmission of the real decisions of participants in the nonblinded approach should also be tested. In our experiment participants working at the first position found 45% of targets. Using a scripted team partner in position 1 of such moderate reliability might in reality lead to a compensatory increase of effort in position 2 and therefore seems worth investigating too. It would also be interesting to examine nonblinded and blinded sequential redundancy in the field, where people might feel less observed and evaluated than in a laboratory study, and SL effects might be larger. Another direction of research could be to investigate the reduction in effort when visual search tasks are assisted by technology, as screeners in mammography and baggage screening are assisted not only by other humans but also by increasingly sophisticated software support. It may seem plausible that the motivation to perform a difficult and tiring visual search is reduced when working with a highly reliable system that achieves high detection standards without "real human" effort as well or perhaps even more.

## Data Availability

Data from both studies are available through the Open Science Framework (OSF) at https://osf.io/vnhx3/?view_only = 2e53f862390b49708a5cfce8e38ea787.
